# 5-Cyclo­pentyl-2-methyl-3-phenyl­sulfonyl-1-benzofuran

**DOI:** 10.1107/S160053681103042X

**Published:** 2011-08-02

**Authors:** Pil Ja Seo, Hong Dae Choi, Byeng Wha Son, Uk Lee

**Affiliations:** a Department of Chemistry, Dongeui University, San 24 Kaya-dong Busanjin-gu, Busan 614-714, Republic of Korea; b Department of Chemistry, Pukyong National University, 599-1 Daeyeon 3-dong, Nam-gu, Busan 608-737, Republic of Korea

## Abstract

In the title compound, C_20_H_20_O_3_S, the cyclo­pentyl ring adopts an envelope conformation. The phenyl ring makes a dihedral angle of 81.40 (6)° with the mean plane of the benzofuran fragment. In the crystal, mol­ecules are linked by weak inter­molecular C—H⋯O hydrogen bonds and C—H⋯π inter­actions.

## Related literature

For the pharmacological activity of benzofuran compounds, see: Aslam *et al.* (2009 ▶); Galal *et al.* (2009 ▶); Khan *et al.* (2005 ▶). For natural products with benzofuran rings, see: Akgul & Anil (2003 ▶); Soekamto *et al.* (2003 ▶). For structural studies of related 5-alkyl-2-methyl-3-phenyl­sulfonyl-1-benzofuran derivatives, see: Choi *et al.* (2008**a* ▶,b* ▶); Seo *et al.* (2011 ▶).
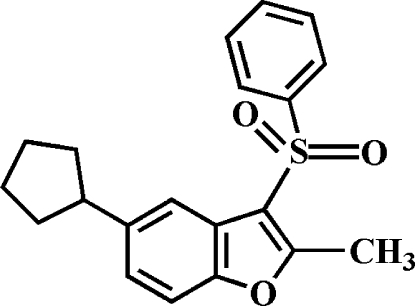


## Experimental

### 

#### Crystal data

C_20_H_20_O_3_S
*M* *_r_* = 340.42
Monoclinic, 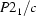

*a* = 6.2999 (9) Å
*b* = 15.001 (2) Å
*c* = 17.743 (2) Å
β = 92.011 (3)°
*V* = 1675.8 (4) Å^3^
*Z* = 4
Mo *K*α radiation
μ = 0.21 mm^−1^
*T* = 173 K
0.27 × 0.10 × 0.08 mm

#### Data collection

Bruker SMART APEXII CCD diffractometer
Absorption correction: multi-scan (*SADABS*; Bruker, 2009 ▶) *T*_min_ = 0.946, *T*_max_ = 0.983
15497 measured reflections
3616 independent reflections
2869 reflections with *I* > 2σ(*I*)
*R*_int_ = 0.065

#### Refinement

*R*[*F* ^2^ > 2σ(*F* ^2^)] = 0.052
*wR*(*F* ^2^) = 0.130
*S* = 1.04
3616 reflections
218 parameters
H-atom parameters constrained
Δρ_max_ = 0.44 e Å^−3^
Δρ_min_ = −0.37 e Å^−3^

### 

Data collection: *APEX2* (Bruker, 2009 ▶); cell refinement: *SAINT* (Bruker, 2009 ▶); data reduction: *SAINT*; program(s) used to solve structure: *SHELXS97* (Sheldrick, 2008 ▶); program(s) used to refine structure: *SHELXL97* (Sheldrick, 2008 ▶); molecular graphics: *ORTEP-3* (Farrugia, 1997 ▶) and *DIAMOND* (Brandenburg, 1998 ▶); software used to prepare material for publication: *SHELXL97*.

## Supplementary Material

Crystal structure: contains datablock(s) global, I. DOI: 10.1107/S160053681103042X/zl2391sup1.cif

Structure factors: contains datablock(s) I. DOI: 10.1107/S160053681103042X/zl2391Isup2.hkl

Supplementary material file. DOI: 10.1107/S160053681103042X/zl2391Isup3.cml

Additional supplementary materials:  crystallographic information; 3D view; checkCIF report

## Figures and Tables

**Table 1:** Hydrogen-bond geometry (Å, °) *Cg* is the centroid of the C1/C2/C7/O1/C8 furan ring.

*D*—H⋯*A*	*D*—H	H⋯*A*	*D*⋯*A*	*D*—H⋯*A*
C12—H12*A*⋯O3^i^	0.99	2.52	3.493 (3)	166
C17—H17⋯O3^ii^	0.95	2.49	3.183 (3)	129
C10—H10*B*⋯*Cg*^iii^	0.99	2.68	3.604 (3)	156

Symmetry codes: (i) 
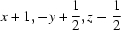
; (ii) 
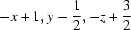
; (iii) 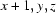.

**Fig. 1.**
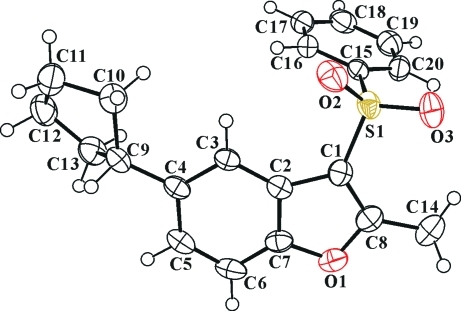
The molecular structure of the title compound with the atom numbering scheme. Displacement ellipsoids are drawn at the 50% probability level. H atoms are presented as small spheres of arbitrary radius.

**Fig. 2.**
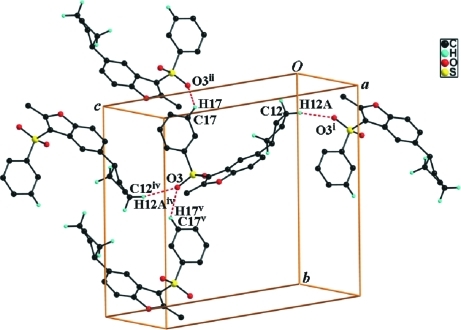
A view of the C—H···O interactions (dotted lines) in the crystal structure of the title compound. [Symmetry codes: (i) x + 1, - y + 1/2, z - 1/2; (ii) - x + 1 , y - 1/2, - z + 3/2; (iv) x - 1, - y + 1/2, z + 1/2; (v) - x + 1, y + 1/2, - z + 3/2.]

**Fig. 3.**
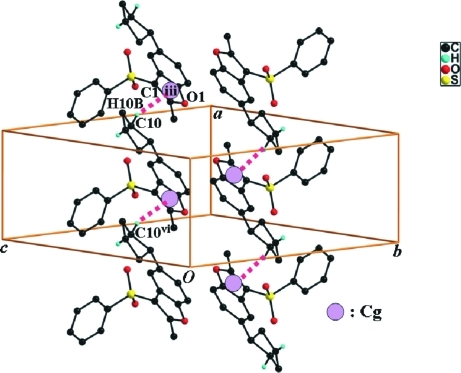
A view of the C—H···π interactions (dotted lines) in the crystal structure of the title compound. [Symmetry codes: (iii) x + 1, y, z; (vi) x - 1, y, z.]

## supplementary crystallographic information

### Comment

Recently, many compounds involving a benzofuran skeleton have drawn much attention owing to their valuable pharmacological properties such as antibacterial and antifungal, antitumor and antiviral, and antimicrobial activities  (Aslam *et al.*, 2009, Galal *et al.*, 2009, Khan *et al.*, 2005). These benzofuran derivatives occur in a wide range of natural products (Akgul & Anil, 2003; Soekamto *et al.*, 2003). As a part of our continuing studies of the substituent effect on the solid state structures of 5-alkyl-2-methyl-3-phenylsulfonyl-1-benzofuran analogues (Choi *et al.*, 2008**a*,b*; Seo *et al.*, 2011), we report herein the crystal structure of the title compound.

In the title molecule (Fig. 1), the benzofuran unit is essentially planar, with a mean deviation of 0.020 (2) Å from the least-squares plane defined by the nine constituent atoms. The cyclopentyl ring is in the envelope form. The dihedral angle formed by the phenyl ring and the mean plane of the benzofuran fagment is 81.40 (6)°. The molecular packing (Fig. 2) is stabilized by weak intermolecular C—H···O hydrogen bonds; the first one between a cyclopentyl H atom and the O atom of the sulfonyl group (Table 1; C12—H12A···O3^i^), and the second one between a phenyl H atom and the O atom of the sulfonyl group (Table 1; C17—H17···O3^ii^). The crystal packing (Fig. 3) is further stabilized by intermolecular C—H···π interactions between a cyclopentyl H atom and the furan ring (Table 1; C10—H10B···Cg^iii^, Cg is the centroid of the C1/C2/C7/O1/C8  furan ring).

### Experimental

77% 3-chloroperoxybenzoic acid (448 mg, 2.0 mmol) was added in small portions to a stirred solution of 5-cyclopentyl-2-methyl-3-phenylsulfanyl-1-benzofuran (277 mg, 0.9 mmol) in dichloromethane (40 mL) at 273 K. After being stirred at room temperature for 8h, the mixture was washed with saturated sodium bicarbonate solution and the organic layer was separated, dried over magnesium sulfate, filtered and concentrated at reduced pressure. The residue was purified by column chromatography (hexane–ethyl acetate, 4:1 v/v) to afford the title compound as a colorless solid [yield 72%, m.p. 418–419 K; *R*f = 0.48 (hexane–ethyl acetate, 4:1 v/v)]. Single crystals suitable for X-ray diffraction were prepared by slow evaporation of a solution of the title compound in acetone at room temperature.

### Refinement

All H atoms were positioned geometrically and refined using a riding model, with C—H = 0.95 Å for aryl, 1.00 Å for methine, 0.99 Å for methylene and 0.98 Å for methyl H atoms, respectively. *U*_iso_(H)  = 1.2*U*_eq_(C) for aryl, methine and methylene, and 1.5*U*_eq_(C) for methyl H atoms.

### Crystal data

**Table:**

C_20_H_20_O_3_S	*F*(000) = 720
*M**_r_* = 340.42	*D*_x_ = 1.349 Mg m^−^^3^
Monoclinic, *P*2_1_/*c*	Mo *K*α radiation, λ = 0.71073 Å
Hall symbol: -P 2ybc	Cell parameters from 3720 reflections
*a* = 6.2999 (9) Å	θ = 2.3–25.9°
*b* = 15.001 (2) Å	µ = 0.21 mm^−^^1^
*c* = 17.743 (2) Å	*T* = 173 K
β = 92.011 (3)°	Block, colourless
*V* = 1675.8 (4) Å^3^	0.27 × 0.10 × 0.08 mm
*Z* = 4	

### Data collection

**Table:**

Bruker SMART APEXII CCD diffractometer	3616 independent reflections
Radiation source: rotating anode	2869 reflections with *I* > 2σ(*I*)
graphite multilayer	*R*_int_ = 0.065
Detector resolution: 10.0 pixels mm^-1^	θ_max_ = 27.0°, θ_min_ = 1.8°
φ and ω scans	*h* = −8→8
Absorption correction: multi-scan (*SADABS*; Bruker, 2009)	*k* = −18→19
*T*_min_ = 0.946, *T*_max_ = 0.983	*l* = −22→22
15497 measured reflections	

### Refinement

**Table:**

Refinement on *F*^2^	Primary atom site location: structure-invariant direct methods
Least-squares matrix: full	Secondary atom site location: difference Fourier map
*R*[*F*^2^ > 2σ(*F*^2^)] = 0.052	Hydrogen site location: difference Fourier map
*wR*(*F*^2^) = 0.130	H-atom parameters constrained
*S* = 1.04	*w* = 1/[σ^2^(*F*_o_^2^) + (0.0491*P*)^2^ + 0.8019*P*] where *P* = (*F*_o_^2^ + 2*F*_c_^2^)/3
3616 reflections	(Δ/σ)_max_ < 0.001
218 parameters	Δρ_max_ = 0.44 e Å^−^^3^
0 restraints	Δρ_min_ = −0.37 e Å^−^^3^

### Special details

**Table:**

Geometry. All esds (except the esd in the dihedral angle between two l.s. planes) are estimated using the full covariance matrix. The cell esds are taken into account individually in the estimation of esds in distances, angles and torsion angles; correlations between esds in cell parameters are only used when they are defined by crystal symmetry. An approximate (isotropic) treatment of cell esds is used for estimating esds involving l.s. planes.
Refinement. Refinement of F^2^ against ALL reflections. The weighted R-factor wR and goodness of fit S are based on F^2^, conventional R-factors R are based on F, with F set to zero for negative F^2^. The threshold expression of F^2^ > 2sigma(F^2^) is used only for calculating R-factors(gt) etc. and is not relevant to the choice of reflections for refinement. R-factors based on F^2^ are statistically about twice as large as those based on F, and R- factors based on ALL data will be even larger.

### Fractional atomic coordinates and isotropic or equivalent isotropic displacement parameters (Å^2^)

**Table:**

	*x*	*y*	*z*	*U*_iso_*/*U*_eq_	
S1	0.49451 (8)	0.34441 (4)	0.69600 (3)	0.03570 (17)	
O1	0.2815 (2)	0.42140 (10)	0.49705 (9)	0.0416 (4)	
O2	0.7200 (2)	0.33502 (12)	0.70766 (9)	0.0469 (4)	
O3	0.3805 (3)	0.40440 (11)	0.74236 (9)	0.0502 (4)	
C1	0.4473 (3)	0.37248 (14)	0.60261 (12)	0.0337 (5)	
C2	0.5648 (3)	0.33976 (13)	0.53954 (11)	0.0311 (4)	
C3	0.7450 (3)	0.28710 (13)	0.53044 (11)	0.0304 (4)	
H3	0.8255	0.2661	0.5731	0.036*	
C4	0.8043 (3)	0.26593 (13)	0.45818 (11)	0.0312 (4)	
C5	0.6835 (3)	0.29847 (15)	0.39625 (12)	0.0391 (5)	
H5	0.7246	0.2829	0.3469	0.047*	
C6	0.5081 (4)	0.35192 (16)	0.40378 (13)	0.0417 (5)	
H6	0.4289	0.3742	0.3613	0.050*	
C7	0.4540 (3)	0.37123 (14)	0.47631 (12)	0.0349 (5)	
C8	0.2785 (3)	0.42026 (14)	0.57351 (13)	0.0380 (5)	
C9	0.9944 (3)	0.20828 (14)	0.44394 (11)	0.0341 (5)	
H9	1.0897	0.2426	0.4106	0.041*	
C10	1.1282 (3)	0.17647 (15)	0.51169 (13)	0.0399 (5)	
H10A	1.0380	0.1504	0.5506	0.048*	
H10B	1.2124	0.2260	0.5343	0.048*	
C11	1.2724 (4)	0.10590 (17)	0.47845 (15)	0.0503 (6)	
H11A	1.3148	0.0610	0.5170	0.060*	
H11B	1.4021	0.1336	0.4589	0.060*	
C12	1.1391 (4)	0.06297 (18)	0.41446 (14)	0.0551 (7)	
H12A	1.2193	0.0619	0.3675	0.066*	
H12B	1.1012	0.0010	0.4277	0.066*	
C13	0.9395 (4)	0.11989 (16)	0.40397 (13)	0.0463 (6)	
H13A	0.8167	0.0908	0.4272	0.056*	
H13B	0.9051	0.1299	0.3498	0.056*	
C14	0.1001 (4)	0.46840 (16)	0.60728 (15)	0.0508 (6)	
H14A	0.1019	0.5309	0.5914	0.076*	
H14B	0.1147	0.4652	0.6624	0.076*	
H14C	−0.0346	0.4410	0.5905	0.076*	
C15	0.3793 (3)	0.23799 (14)	0.70462 (11)	0.0306 (4)	
C16	0.4923 (4)	0.16333 (15)	0.68364 (12)	0.0397 (5)	
H16	0.6306	0.1693	0.6645	0.048*	
C17	0.4017 (4)	0.08023 (16)	0.69086 (13)	0.0484 (6)	
H17	0.4779	0.0285	0.6769	0.058*	
C18	0.2011 (4)	0.07225 (16)	0.71818 (13)	0.0475 (6)	
H18	0.1400	0.0148	0.7235	0.057*	
C19	0.0885 (4)	0.14626 (16)	0.73774 (13)	0.0438 (5)	
H19	−0.0504	0.1399	0.7562	0.053*	
C20	0.1760 (3)	0.23023 (15)	0.73080 (12)	0.0378 (5)	
H20	0.0977	0.2818	0.7438	0.045*	

### Atomic displacement parameters (Å^2^)

**Table:**

	*U*^11^	*U*^22^	*U*^33^	*U*^12^	*U*^13^	*U*^23^
S1	0.0368 (3)	0.0405 (3)	0.0297 (3)	−0.0009 (2)	−0.0001 (2)	−0.0102 (2)
O1	0.0392 (8)	0.0379 (9)	0.0475 (10)	0.0027 (6)	−0.0034 (7)	0.0097 (7)
O2	0.0366 (8)	0.0688 (12)	0.0350 (9)	−0.0052 (7)	−0.0044 (6)	−0.0048 (8)
O3	0.0600 (10)	0.0460 (10)	0.0449 (10)	−0.0011 (8)	0.0077 (8)	−0.0213 (8)
C1	0.0347 (10)	0.0302 (10)	0.0361 (12)	−0.0023 (8)	0.0001 (9)	−0.0050 (9)
C2	0.0348 (10)	0.0296 (10)	0.0288 (11)	−0.0066 (8)	−0.0008 (8)	−0.0004 (8)
C3	0.0339 (10)	0.0318 (11)	0.0253 (10)	−0.0038 (8)	−0.0027 (8)	0.0015 (8)
C4	0.0347 (10)	0.0326 (11)	0.0262 (11)	−0.0109 (8)	0.0002 (8)	0.0023 (8)
C5	0.0450 (12)	0.0464 (13)	0.0260 (11)	−0.0102 (10)	0.0021 (9)	0.0043 (9)
C6	0.0433 (12)	0.0494 (14)	0.0318 (12)	−0.0079 (10)	−0.0067 (9)	0.0155 (10)
C7	0.0335 (10)	0.0320 (11)	0.0390 (12)	−0.0040 (8)	−0.0031 (9)	0.0077 (9)
C8	0.0380 (11)	0.0294 (11)	0.0465 (14)	−0.0032 (8)	−0.0009 (9)	−0.0003 (9)
C9	0.0387 (11)	0.0360 (11)	0.0280 (11)	−0.0076 (9)	0.0071 (9)	0.0003 (9)
C10	0.0380 (11)	0.0415 (13)	0.0402 (13)	−0.0051 (9)	−0.0004 (9)	−0.0068 (10)
C11	0.0436 (13)	0.0509 (15)	0.0569 (16)	0.0045 (11)	0.0083 (11)	−0.0020 (12)
C12	0.0709 (17)	0.0497 (15)	0.0459 (15)	0.0079 (12)	0.0178 (13)	−0.0066 (12)
C13	0.0574 (14)	0.0453 (14)	0.0360 (13)	−0.0031 (11)	−0.0001 (11)	−0.0090 (10)
C14	0.0413 (12)	0.0395 (13)	0.0715 (18)	0.0065 (10)	0.0017 (12)	−0.0011 (12)
C15	0.0343 (10)	0.0370 (11)	0.0201 (10)	0.0053 (8)	−0.0036 (8)	−0.0044 (8)
C16	0.0434 (12)	0.0450 (13)	0.0310 (12)	0.0156 (10)	0.0062 (9)	0.0021 (9)
C17	0.0731 (16)	0.0380 (13)	0.0345 (13)	0.0196 (11)	0.0065 (11)	0.0013 (10)
C18	0.0697 (16)	0.0392 (13)	0.0332 (13)	−0.0023 (11)	−0.0015 (11)	−0.0002 (10)
C19	0.0406 (12)	0.0512 (14)	0.0393 (13)	−0.0034 (10)	−0.0004 (10)	−0.0015 (10)
C20	0.0360 (11)	0.0413 (12)	0.0361 (12)	0.0076 (9)	0.0005 (9)	−0.0070 (9)

### Geometric parameters (Å, °)

**Table:**

S1—O3	1.4295 (15)	C10—H10B	0.9900
S1—O2	1.4351 (16)	C11—C12	1.530 (4)
S1—C1	1.725 (2)	C11—H11A	0.9900
S1—C15	1.763 (2)	C11—H11B	0.9900
O1—C8	1.358 (3)	C12—C13	1.526 (3)
O1—C7	1.382 (3)	C12—H12A	0.9900
C1—C8	1.368 (3)	C12—H12B	0.9900
C1—C2	1.449 (3)	C13—H13A	0.9900
C2—C7	1.383 (3)	C13—H13B	0.9900
C2—C3	1.397 (3)	C14—H14A	0.9800
C3—C4	1.385 (3)	C14—H14B	0.9800
C3—H3	0.9500	C14—H14C	0.9800
C4—C5	1.402 (3)	C15—C20	1.383 (3)
C4—C9	1.506 (3)	C15—C16	1.385 (3)
C5—C6	1.376 (3)	C16—C17	1.379 (3)
C5—H5	0.9500	C16—H16	0.9500
C6—C7	1.374 (3)	C17—C18	1.374 (4)
C6—H6	0.9500	C17—H17	0.9500
C8—C14	1.481 (3)	C18—C19	1.369 (3)
C9—C10	1.520 (3)	C18—H18	0.9500
C9—C13	1.537 (3)	C19—C20	1.382 (3)
C9—H9	1.0000	C19—H19	0.9500
C10—C11	1.527 (3)	C20—H20	0.9500
C10—H10A	0.9900		
			
O3—S1—O2	119.56 (10)	C10—C11—C12	105.06 (19)
O3—S1—C1	109.02 (10)	C10—C11—H11A	110.7
O2—S1—C1	107.38 (10)	C12—C11—H11A	110.7
O3—S1—C15	107.67 (10)	C10—C11—H11B	110.7
O2—S1—C15	107.89 (10)	C12—C11—H11B	110.7
C1—S1—C15	104.29 (10)	H11A—C11—H11B	108.8
C8—O1—C7	107.31 (16)	C13—C12—C11	106.46 (19)
C8—C1—C2	107.29 (19)	C13—C12—H12A	110.4
C8—C1—S1	126.56 (17)	C11—C12—H12A	110.4
C2—C1—S1	125.71 (16)	C13—C12—H12B	110.4
C7—C2—C3	119.22 (19)	C11—C12—H12B	110.4
C7—C2—C1	104.71 (18)	H12A—C12—H12B	108.6
C3—C2—C1	136.06 (19)	C12—C13—C9	104.8 (2)
C4—C3—C2	118.90 (19)	C12—C13—H13A	110.8
C4—C3—H3	120.5	C9—C13—H13A	110.8
C2—C3—H3	120.5	C12—C13—H13B	110.8
C3—C4—C5	119.3 (2)	C9—C13—H13B	110.8
C3—C4—C9	121.93 (18)	H13A—C13—H13B	108.9
C5—C4—C9	118.78 (18)	C8—C14—H14A	109.5
C6—C5—C4	122.9 (2)	C8—C14—H14B	109.5
C6—C5—H5	118.6	H14A—C14—H14B	109.5
C4—C5—H5	118.6	C8—C14—H14C	109.5
C7—C6—C5	116.1 (2)	H14A—C14—H14C	109.5
C7—C6—H6	121.9	H14B—C14—H14C	109.5
C5—C6—H6	121.9	C20—C15—C16	120.9 (2)
C6—C7—O1	126.0 (2)	C20—C15—S1	119.64 (16)
C6—C7—C2	123.6 (2)	C16—C15—S1	119.45 (16)
O1—C7—C2	110.42 (18)	C17—C16—C15	119.2 (2)
O1—C8—C1	110.25 (19)	C17—C16—H16	120.4
O1—C8—C14	115.8 (2)	C15—C16—H16	120.4
C1—C8—C14	133.9 (2)	C18—C17—C16	120.0 (2)
C4—C9—C10	118.02 (17)	C18—C17—H17	120.0
C4—C9—C13	113.90 (17)	C16—C17—H17	120.0
C10—C9—C13	101.75 (18)	C19—C18—C17	120.6 (2)
C4—C9—H9	107.5	C19—C18—H18	119.7
C10—C9—H9	107.5	C17—C18—H18	119.7
C13—C9—H9	107.5	C18—C19—C20	120.3 (2)
C9—C10—C11	103.48 (18)	C18—C19—H19	119.8
C9—C10—H10A	111.1	C20—C19—H19	119.8
C11—C10—H10A	111.1	C19—C20—C15	118.9 (2)
C9—C10—H10B	111.1	C19—C20—H20	120.5
C11—C10—H10B	111.1	C15—C20—H20	120.5
H10A—C10—H10B	109.0		
			
O3—S1—C1—C8	−21.6 (2)	S1—C1—C8—O1	−173.51 (15)
O2—S1—C1—C8	−152.50 (19)	C2—C1—C8—C14	178.7 (2)
C15—S1—C1—C8	93.2 (2)	S1—C1—C8—C14	6.0 (4)
O3—S1—C1—C2	167.01 (17)	C3—C4—C9—C10	−2.7 (3)
O2—S1—C1—C2	36.1 (2)	C5—C4—C9—C10	177.77 (18)
C15—S1—C1—C2	−78.21 (19)	C3—C4—C9—C13	116.6 (2)
C8—C1—C2—C7	−0.1 (2)	C5—C4—C9—C13	−63.0 (2)
S1—C1—C2—C7	172.67 (15)	C4—C9—C10—C11	168.43 (18)
C8—C1—C2—C3	−178.6 (2)	C13—C9—C10—C11	43.0 (2)
S1—C1—C2—C3	−5.8 (3)	C9—C10—C11—C12	−32.8 (2)
C7—C2—C3—C4	−1.7 (3)	C10—C11—C12—C13	9.5 (3)
C1—C2—C3—C4	176.6 (2)	C11—C12—C13—C9	17.1 (3)
C2—C3—C4—C5	0.6 (3)	C4—C9—C13—C12	−165.15 (18)
C2—C3—C4—C9	−178.99 (17)	C10—C9—C13—C12	−37.1 (2)
C3—C4—C5—C6	0.8 (3)	O3—S1—C15—C20	19.5 (2)
C9—C4—C5—C6	−179.64 (19)	O2—S1—C15—C20	149.86 (17)
C4—C5—C6—C7	−0.9 (3)	C1—S1—C15—C20	−96.18 (18)
C5—C6—C7—O1	−178.17 (19)	O3—S1—C15—C16	−161.71 (17)
C5—C6—C7—C2	−0.3 (3)	O2—S1—C15—C16	−31.40 (19)
C8—O1—C7—C6	176.6 (2)	C1—S1—C15—C16	82.56 (18)
C8—O1—C7—C2	−1.5 (2)	C20—C15—C16—C17	−1.6 (3)
C3—C2—C7—C6	1.6 (3)	S1—C15—C16—C17	179.70 (17)
C1—C2—C7—C6	−177.2 (2)	C15—C16—C17—C18	0.4 (3)
C3—C2—C7—O1	179.78 (16)	C16—C17—C18—C19	0.7 (4)
C1—C2—C7—O1	1.0 (2)	C17—C18—C19—C20	−0.5 (4)
C7—O1—C8—C1	1.4 (2)	C18—C19—C20—C15	−0.7 (3)
C7—O1—C8—C14	−178.18 (18)	C16—C15—C20—C19	1.7 (3)
C2—C1—C8—O1	−0.8 (2)	S1—C15—C20—C19	−179.54 (17)

### Hydrogen-bond geometry (Å, °)

**Table:**

Cg is the centroid of the C1/C2/C7/O1/C8 furan ring.

**Table:**

*D*—H···*A*	*D*—H	H···*A*	*D*···*A*	*D*—H···*A*
C12—H12A···O3^i^	0.99	2.52	3.493 (3)	166.
C17—H17···O3^ii^	0.95	2.49	3.183 (3)	129.
C10—H10B···Cg^iii^	0.99	2.68	3.604 (3)	156.

Symmetry codes: (i) *x*+1, −*y*+1/2, *z*−1/2; (ii) −*x*+1, *y*−1/2, −*z*+3/2; (iii) *x*+1, *y*, *z*.
